# Validity and Reliability of a Wearable Fitness Technology Scale in Portugal

**DOI:** 10.3390/ijerph19105927

**Published:** 2022-05-13

**Authors:** Vera Pedragosa, Salvador Angosto, Celina Gonçalves

**Affiliations:** 1 Research Center in Business and Economics (CICEE), Department of Economic and Business Sciences, Universidade Autónoma de Lisboa, 1169-023 Lisbon, Portugal; 2 Psychology Research Centre (CIP), Department of Economic and Business Sciences, Universidade Autónoma de Lisboa, 1169-023 Lisbon, Portugal; 3 Department of Physical Activity and Sport, Faculty of Sport Sciences, University of Murcia, 30100 Murcia, Spain; salvador.a.s@um.es; 4 Department of Physical Education and Sport, Faculty of Educational Sciences, University of Seville, 41013 Seville, Spain; 5 Research Centre in Sports Sciences, Health Sciences and Human Development (CIDESD), Universidade da Maia (UMaia), 4475-690 Maia, Portugal; celinag@ismai.pt; 6 Instituto Politécnico de Bragança (IPB), 5300-253 Bragança, Portugal

**Keywords:** members’ perceptions, users, Wearable Fitness Technology, fitness centres, validation

## Abstract

Currently, the use of technological devices for monitoring physical activity and in other mobile applications is widespread among sports users and is continuously growing. The aim of this study was the validation of the reliability of the use of Wearable Fitness Technology (WFT) in the context of fitness through quantitative analysis. Data collection was conducted online during the COVID-19 pandemic period. The sample consisted of 177 members of fitness centres who used MYZONE technology in Portugal. An Exploratory Factor Analysis (EFA) (Factor v10) and a Confirmatory Factor Analysis (CFA) (AMOS v22.0) were used to test the item fit. The results showed adequate fits, identifying a total of 14 items in a single factor to assess WFT in MYZONE users in fitness centres. The reliability of the scale showed adequate indices within the indicated limits. This study extends the current literature on WFT; provides information for providers, managers, and members; and aims to improve the fitness experience by developing a valid and reliable tool to assess the characteristics and incidence of WFT in fitness centres. The complexity of the WFT will affect the degree of user engagement with the fitness centre, highlighting the importance of using staff skills to generate motivational and innovative challenges to improve the service experience. Furthermore, this scale could be used to examine the influences of WFT on managers’ and members’ perceptions of the service experience in the Portuguese context.

## 1. Introduction

The fitness industry has been continuously growing around the world, according to the latest International Health, Racquet & Sportsclub Association (IHRSA) [1] report, which identifies a total of 184 million members, 205,000 fitness clubs (i.e., gyms, fitness centres), and €81.7 billion of annual revenue worldwide. In Europe, the number of fitness centres stands at 63.3, with 64.8 million members and €28.2 billion in annual revenue [2]. In 2019, the Portuguese fitness industry reached 688,210 members across 1100 gyms, and the global market represented €289.371 million [3]. In 2020, the COVID-19 pandemic had a negative impact on the world in general and Portugal’s fitness industry specifically. The number of fitness clubs decreased by 30% to 800 and the number of members decreased by 29% (491,355 members, 53% of which are women). The global market revenue decreased by 42% to €167,408 million [4]. Coupled with the market downturn, due to COVID-19, the number of innovative technology-based services have increased; these consider members’ satisfaction and positive behavioural intentions (i.e., to continue paying and attending a gym) [5]. Thus, fitness centres have invested in technologically based innovation [6]: applications (apps); virtual classes (in the gym and/or through digital platforms—for instance, Zoom); WFT; and On-demand (Od). The term fitness apps refers to software developed for electronic devices such as smartphones and tablets created for the management of fitness centres—for instance, booking classes and purchasing services [7]. WFTs are electronic devices that can be used to monitor members’ physical activity and set goals, and include games and competitions [8]. Od offers, through digital platforms, various fitness services (i.e., pre-recorded group lessons) that members can use anywhere, according to their needs. As a rule, WFT and Od have an aggregated use price. Johnson [9] refers to wearable technology as a billion-dollar business with growth expected in this area, which is rooted in multiple levels of athletic competition.

There is increasing evidence that fitness technologies, such as apps, WFT, virtual classes, and Od, create opportunities for fitness organisations to improve their service experience [7]. The latest global fitness trends place these applications in the top 20 of all applications, revealing a trend of growth, with these apps moving from the 25th position in 2020 to the 12th position in 2021 [10]. In Portugal, 66% of fitness centres have apps, 65% have virtual classes, and 20% have WFT [4]. In this sense, fitness centres are looking for ways to differentiate themselves and gain a competitive advantage [11,12].

According to the research of Pizzo et al. [13], based on qualitative analyses in which the influence of WFT (using MYZONE technology) on employees’ and members’ perceptions of service experience was examined, service experience could be enhanced via increased social interaction, gamification, and accountability. The use of WFT within scientific literature is receiving attention, with the intention to use being assessed using the extended ‘Unified Theory of Acceptance and Use of Technology’ (UTAUT2) model, demonstrating that facilitating conditions and habitus are the most influential factors in the continued use of WFT [14]. A recent study explored the drivers behind sports technology use and found differences according to sport type (dynamic/non-dynamic) and motivation (intrinsic or extrinsic) regarding sports technology use, where the perceived characteristics of the technology mediate between context and intention to use [15].

Furthermore, Windasari et al. [16] assessed the effects of actors’ interactions on the continued use of a professional training programme, focusing on dietitians’ choices and participation. Their results highlighted the positive and significant effect of dietitians’ involvement on users’ intention to continue using WFT. In addition, self-efficacy in health management was also found to play a key role in moderating the effect of choice on intention to continue using the programme and WFT. Jung and Kang [17] assessed self-determination in WFT from an age-mediated perspective. The authors considered the frequency of use of the device to assess its influence on the needs for competence, autonomy, and enjoyment, with autonomy being found to be the only influential factor for enjoyment when using WFT. Other previous studies that have considered WFT have done so from the perspective of its relationship to increased physical activity as a means of using the device to quantify exercise performed [18,19]. A literature review by Cho et al. [20] showed that the use of the WFT allows for increased engagement in physical activity. Additionally, these authors highlight the importance of gamification and social incentives as influential elements in such engagement in physical activity.

Yoshida [21] mentioned in his research that the number of studies that refer to social interaction between consumers in the sports context has been growing in recent decades, revealing the importance of the concept. Pizzo et al. [13] concluded that MYZONE increases the social interaction between members, both within and outside of the fitness centre, developing a sense of community between staff and members. In Johnson’s study [9], the results suggest that gamification methods are effective in acquiring, engaging, and retaining individuals, leading to improved athletic performance. According to the results of the study, the research identified a strong positive correlation between gamification and effective motivational strategies for athletes. Specifically, attraction, engagement, and retention are important as athletes progress and lose interest in the traditional structure of sports. Managers need to introduce technology to increase the interest of members. Pizzo et al. [13] concluded that MYZONE helps fitness industry professionals to use gamification to incorporate game-like design elements (e.g., fitness challenges).

The communication, frequency, and technical quality of professionals’ interactions are relevant factors in identifying the satisfaction of members and the quality of fitness centres [22]. Pizzo et al. [13] further reported the importance of monitoring member activity outside the facility to help stimulate additional dialogue between fitness industry professionals and members, thereby considering their practice behaviour. Therefore, the aim of this study was to follow Pizzo et al.’s [13] study, testing the validity and reliability (internal consistency) of the use of the Wearable Fitness Technology Scale (WFTS) in Portugal. This approach will allow the validation of a scale that can be applied in fitness centres, promoting its potential in the fitness industry, as it can help managers improve their services and increase profitability.

## 2. Materials and Methods

### 2.1. Sample

The participants consisted of a total of 177 members of fitness centres, 54.8% female and 45.2% male, with an average age of 39.9 ± 8.9 years. Almost half of the members had more than 10 years of experience in sport (47.0%). Regarding the length of time spent in the fitness centre, 42.2% of the members indicated that they had been a member of a fitness centre for more than three years, with attendance varying between 61 to 90 min a day (41.0%) and with a frequency of 3 days a week (30.0%).

### 2.2. Instrument Development

The WFTS is based on Pizzo et al.’s [13] research and assesses 3 dimensions of 16 items in relation to the users of MYZONE: the social interaction dimension (4 items) aims to assess the sense of community and engagement with the fitness centre; the gamification dimension (6 items) aim to attract, engage, and retain an athlete as they progress; and the accounting dimension (6 items) aims to understand and monitor member activity both inside and outside the fitness centre. MYZONE uses a chest band that offers a platform supporting fitness centre operations. It is promoted and sold by fitness centre professionals (i.e., owners and employees) directly to members. Fitness centres support the integration of the MYZONE platform through wireless technology to monitor members’ physical activity, heart rate, calories burned, and time of exercise. This information is then converted into a point system called MYZONE Effort Points. Fitness centres can create physical activity reports from data measured by the MYZONE device. Additionally, MYZONE users have the option to display a real-time live tile on TV screens in the fitness centres that project the user’s nickname, effort expenditure (for instance, percentage of maximum heart rate), heart rate, calories burned, and points. Each live tile offers a coloured display corresponding to the user’s heart rate zone to be used with heart rate-based training [23].

After these procedures, the final questionnaire includes demographic questions and a pool of 18 items plus 2 item suggestions for professionals of 2 fitness centres distributed among the 3 dimensions related to WFT: 4 items for social interaction; 7 items for gamification; and 7 items for accountability. All items are measured on a 7-point scale ranging from ‘Strongly Disagree’ (1) to ‘Strongly Agree’ (7).

### 2.3. Procedures

All the items proposed by Pizzo et al. [13] were translated into Portuguese. The sport management research authors conducted a content analysis of the items and adapted the main quantitative results by transforming them into statements that could be evaluated quantitatively by means of a Likert scale. A back translation process was undertaken [24]. Table A1 of Appendix A shows the conclusions drawn from the study conducted by Pizzo et al. [13] and the items developed for each according to the opinion of sports managers and experts. First, the items were translated into Portuguese by two bilingual sports management authors. Then, they were reviewed by a professional academic translator, who did not have access to the original English version. Next, they were translated back into English by a Portuguese native sport management author, who is an academic with experience in translations. Finally, the two versions were compared, and we concluded that the instruments were equivalent. The next step was an interview conducted between the authors and the employees of 2 fitness centres which used MYZONE. The results of this meeting led to a linguistic adjustment of some items to the context and 2 items were incorporated in addition to the initial 16 items proposed by Pizzo et al. [13]; these included 1 more item on gamification and 1 more item on accountability.

After the questionnaire items were finalised, the questionnaire was given to the members of the Gimnica database (MYZONE representative company in Portugal) and 2 fitness centres. For this purpose, the survey tool “Lime Survey” from the local university was used. The researchers sent the link of the questionnaire to the managers of Gimnica and the fitness centres, who disseminated it to all their members. The data collection period was between April and May 2021. The sample used was non-probabilistic by convenience, according to the degree of accessibility of the sample [25]. The survey was completely anonymous. Finally, the data were extracted and analysed. 

### 2.4. Data Analysis

First, the psychometric properties and correlation of the scale indicators were tested using the statistical software for social sciences SPSS v.24. Subsequently, the validity of the factor structure was tested using EFA and CFA. The EFA was performed with the FACTOR v.10 programme following the recommendations of Lloret-Segura et al. [26]. This analysis was carried out through the method of extraction of Exploratory Robust Maximum Likelihood (RML); the Oblimin Direct rotation method was also used. To determine the number of factors, the procedure of Optimal Implementation of Parallel Analysis [27] was used and to check the fit of the model. Additionally, the coefficients of root mean square root of the residuals (RMSR) were analysed, as well as the gamma index or the goodness of fit (GFI), as proposed by Tanaka and Huba [28]. Other indicators that were considered were the Generalized G–H Index, which was used to analyse the replicability of the factors derived from the EFA. Kaiser–Meyer–Olkin’s (KMO) measures for sample adequation were also observed, as was Bartlett’s Test of Sphericity (BTS). On the contrary, the items with factorial loads below 0.40 or above this value in two or more factors were eliminated from further analyses. Finally, the theoretical interpretability of the factorial solution extracted from the EFA was tested.

A CFA was carried out by applying the method of Robust Maximum Likelihood Estimation (MLR), with the aim of correcting for the possible absence of multivariate normality using statistics such as the χ^2^ of Satorra–Bentler [29]. Thus, for the evaluation of global fit, different goodness of fit indices recommended in the literature [30] were used, such as the Chi-squared significance and its robust correction offered by Satorra–Bentler (S–B χ^2^) [31]. In addition, other coefficients were calculated, which allowed the adequacy of the proposed models to be tested, such as the ratio of χ^2^ and its degrees of freedom (χ^2^/df), with acceptable values being less than five [32]. In the same way, the coefficients of indices for the robust goodness of fit of the proposed model, such as the Non-Normed Fit Index (NNFI), Compared Fit Index (CFI), Tucker–Lewis Index (TLI), and the Incremental Fit Index (IFI), were tested. For these indicators, a good fit is considered to be represented by a value above 0.90 [33]. After this, the Root Mean Square Error of Approximation (RMSEA) was calculated, with a score below 0.08 considered to be a good fit [34]. Finally, for the evaluation of the reliability of the scales, three measurements were considered: Cronbach’s Alpha (C-α), McDonald’s Omega (ω), Composite Reliability (CR), and the Average Variance Extracted (AVE) for each factor [35,36].

## 3. Results

### 3.1. Analysis of the Psychometric Properties of the Items

To check the psychometric properties of the scale indicators, the values of the mean, standard deviation, skewness, and kurtosis were analysed. This information is shown in Table 1. In general, all items had scores above five points, except for item 10, which had a mean of 4.34 ± 1.8. According to Chou and Bentler [37], the acceptable limit of skewness and kurtosis is an absolute value of 3.0, which was not exceeded by any of the indicators. Correlation analysis (Table 2) showed that all items were statistically significantly related. 

### 3.2. Exploratory Factor Analysis

Table 3 shows the results of the EFA of the WFTS. The 18 chosen items were analysed, and a single factor composed of 14 items was extracted, eliminating 4 items (Item 4, Item 9, Item 16, and Item 17) from the initial model that best fit the objectives of the study. Following the above criteria, four WFTS items with factor loadings below 0.40 or above 0.40 for two or more factors were eliminated. Bartlett’s Test of Sphericity showed significant values and the KMO had good values (BTS (df) = 1623.7(91); *p* ≤ 0.001; KMO = 0.93).

To check the fit of the models, the residual Root Mean Square Root (RMSR) coefficients, the gamma index or GFI, and the comparative fit index or CFI were analysed (RMCR < 0.05; GFI > 0.95; CFI > 0.95). WFT obtained adequate values in the GFI and CFI indices; however, the RMSR index was very close to the ideal value (RMSR = 0.06; GFI = 0.98; CFI = 0.98). In contrast, the Generalised G-H Index showed values above 0.80 (G-H Index = 0.95), indicating the good replicability of the dimensions in other studies [38]. The variance explained by the single WFTS factor was 63.06%. 

### 3.3. Confirmatory Factor Analysis and Reliability

**Figure 1:**
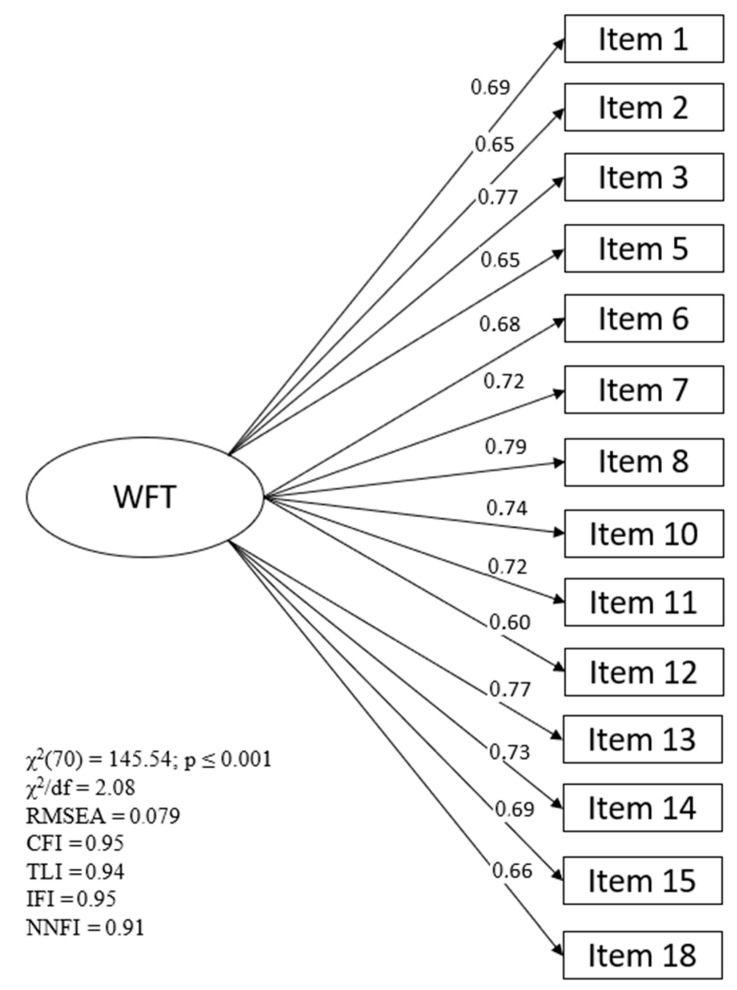
Confirmatory Factor Analysis results.

CFA was used to examine the extent of the latent factors extracted from EFA and determine if they could be validly replicated. The model was tested using the maximum likelihood parameter estimation method. The results of the CFA of WFTS showed that the model adjusted appropriately (χ^2^(70) = 145.54; *p* ≤ 0.001). The standardised Chi-square (χ^2^/df) obtained a value of 2.08, although the ideal range was between 2.0 and 3.0, Bollen [39] indicates that scores up to 5.0 are acceptable. The RMSEA index showed a value of 0.079, lower than 0.08. Along the same lines, the rest of the indices showed a good fit for the model, as they presented values higher than 0.90: NNFI = 0.91; CFI = 0.95; TLI = 0.94; and IFI = 0.95. The CFA load weights of all items of WFT (Figure 1) were adequate. The loads complied with the minimum score recommended (>0.5) and reproduced the initial structure of EFA. Finally, the reliability of the scale showed adequate values, with C-α = 0.93, CR = 0.93, and ω = 0.77 being above the cut-off point of 0.7, while the AVE obtained a value of 0.50, placing it at the minimum recommended value [35,36,40]. 

## 4. Discussion

The aim of the current study was to test the validity and reliability of the Wearable Fitness Technology Scale (WFTS) through a sample of Portuguese fitness members. WFT is a concept introduced in recent years and is an emerging line of research, with only a limited number of studies available. This study developed an initial quantitative scale that assesses WFT in the fitness sector based on the qualitative study developed by Pizzo et al. [13]. 

The results of the validation of this scale indicated that the initial 18 items used could be reduced to 14 items, as items 4, 9, 16, and 17 did not adequately saturate and only one factor was extracted from the EFA. Concerning the removed items, these were: “Excessive sharing of personal WFT physical activity data” (social interaction dimension); “Changes to the physical design and layout of fitness facilities to support the use of WFT” (gamification dimension); “Fitness facility’s and staff’s monitoring of WFT users´ activity beyond the physical facility” (accountability dimension); and “Fitness facility’s and staff’s use of WFT data to reduce/limit users’ physical activity” (accountability dimension). 

The EFA was conducted following the recommendations suggested by Lloret-Segura et al. [26], and the indices, such as the GFI, RMSR, and G-H, showed satisfactory values above the cut-off points established by the literature [28,38]. However, the results showed that all the items fitted into a single factor (WFT) and not into the three dimensions of social interaction, gamification, and accounting proposed by Pizzo et al. [13]. 

There may be several reasons for these results; however, it should be noted that this study was carried out while fitness centres were closed, from 13 January to 5 May 2021 [4], as the world was in the middle of a pandemic, with lower adherence to gyms, less physical activity, and lower retention rates (2%) than could be expected usually. These circumstances made it difficult to include a large number of centres using the WFT; thus, we were unable to comply with the ratio for the number of participants per item recommended by MacCallum et al. [41].

Furthermore, the results of the CFA showed that the validity and reliability were adequate to replicate this study at a pilot scale in fitness settings that have implemented the WFTS in their facilities. All fit indices were satisfactory, except for TLI, which showed a value close to 0.90 [30]. The C-α, CR, and AVE were satisfactory, with values well above the 0.90 cut-off points for NNFI, TLI, and IFI [30,33], while the CFI was above 0.95 [42]. Finally, the reliability indices showed values above 0.70 for C-α, CR, and ω [35,36,40], with the value for AVE being at the 0.50 cut-off point [35]. Finally, in contrast to Pizzo et al.’s [13] work, our findings suggest only one dimension, with 14 items, was perceived to improve service experience, without conceptualising social interaction, gamification, and accountability. 

The majority of members identified the items chosen as relevant to their sports experience with Wearable Fitness Technology, which is in line with the findings of other studies [43,44]. Our findings revealed the importance of one dimension with 14 items and showed the perceptions of members regarding the functionality of WFT-MYZONE. In line with other studies, our results show increased member engagement in relation to staff and fitness centres [12]. Specifically, our results demonstrate that the most relevant items were “An increase in the sense of community among MYZONE members and fitness facility staff”; “Addition of competitive elements to physical activity based on MYZONE member data”; and “Quantified physical activity metrics of MYZONE members”. The appreciation of these items by the members supports the research already conducted in the fitness industry, which highlights the importance of the interaction between staff and members to improving the satisfaction of members [45]. The WFT was integrated into a new innovative fitness service that is important for the quality of fitness centres, members’ satisfaction, and member retention [5]. Furthermore, this competitive advantage, nowadays, is supported by technological innovations that are a source of satisfaction for new and existing members [7].

### 4.1. Limitations and Future Research

This study has some limitations that should be considered in future studies. The main limitation of this study is its small sample size. On the one hand, the small size of the sample may be due to the fact that the data collection period occurred during a pandemic, when Portugal’s fitness centres were not typically operating. The Portuguese government, due to the COVID-19 pandemic, temporarily closed fitness centres or reduced their capacity for members when the number of cases was high. Thus, it is likely that the willingness of many members to respond was affected by these circumstances. On the other hand, the number of fitness centres that had implemented WFT in Portugal was very limited, meaning the target population was small. 

In turn, the convenience sampling method used was limited by the researchers’ access to the members of fitness centres and the database of Gimnica; thus, the results obtained cannot be generalised. In addition, a small sample size limits the adequate performance of the factor analysis and prevents the initial structure of three factors from being replicated. Reliability data should be treated with caution, as the EFA extracted a single factor with many items.

Regarding future avenues of research, in order to develop this study further, it would be appropriate to carry out adequate sampling, taking into account members’ gender, culture, and country of origin, as well as different types of fitness centres, to allow the generalisation of the findings. Furthermore, MYZONE is a WFT that is used around the chest, and there are other WFTs that can be used around the wrist (for instance, Fitbit devices). However, chest-based wearables generally require support from fitness centres (for instance, they can be used in fitness centres and also at home). 

Future research should consider the use of better sampling to obtain a sufficient sample in order to replicate this study and analyse the initial structure proposed according to the results of Pizzo et al. [13]. It is also important to point out that it is likely that, in a new data collection period, fitness centres will operate in a more normal way and members will be able to use WFT conveniently. Beyond this, future research should be supported by establishing relations between WFT and satisfaction, retention, and members’ lifetime in order to improve the profitability of fitness centres.

### 4.2. Managerial Implications

Fitness centres and staff (e.g., managers, personal trainers, and technical professionals) should integrate MYZONE or other similar WFT to improve users’ fitness experience. In general, connection and engagement with the members depend on the complexity of WFT has and the skills of the staff working with the equipment. In this sense, it is important that fitness centres and staff explore the potential of WFTs and have the skills to improve, for instance, the challenges available to members or other situations that can engage them.

However, the results of this study can be incorporated into questionnaires, such as those often carried out by fitness centre managers, to assess members’ perceptions of the quality of and satisfaction with the use of WFT MYZONE. Satisfaction and quality scales have been widely disseminated and studied in fitness, and a major concern of managers is how to improve quality and satisfaction in order to increase members’ loyalty [7,15]. The results of these questionnaires, which reflect the perceptions of gym members, will make it possible to improve the services offered and the parameterisation of the WFT. For instance, members provided direct, first-hand insight into their experience, whereas managers and instructors provided indirect perspectives on managing consumer experiences with WFT.

Beyond this, this scale can be used by other WFTs as long as they have similar characteristics. However, if they are not similar, the characteristics of this WFT MYZONE may serve as a support for suppliers aiming to develop WFTs for the market. Suppliers of this type of technology should also know what kind of parameterisation is important in order to improve the experience of members. 

## 5. Conclusions

This is the first study that has carried out the validation of the reliability of a scale for Wearable Fitness Technology (WFT) using the MYZON technology in Portugal through quantitative analysis. The results suggest the usefulness of the construction of an instrument with the ability to assess members’ perception of technology. However, taking into account that we used convenience sampling limited to fitness centres and the Gymnica database, as well as a single factor with 14 items that fit the objectives of this study, these items can explain only 63% of the variance.

Objectively, the majority of members identified the items as relevant to their experience with WFT. Interestingly, the following items were not considered relevant in this study: changes in the physical design and layout of facilities; the excessive sharing of personal data from physical activities; the monitoring of users’ activity outside of physical facilities; and using data to reduce or limit users’ physical activity. 

With this purpose in mind, this scale should be considered as a means to examine the influences of WFT on employees’ and members’ perceptions of the service experience. 

WFT offers fitness centres the opportunity to improve fitness services, transforming the routine of training into a positive emotional experience that leads to higher levels of satisfaction among members [46]. Nevertheless, WFT can create additional opportunities for fitness centres to engage deeply with their members. WFT is considered a fitness market trend and should be aligned with the expectations of its members [47]. 

One of the strengths of this study is its focus on WFT—in particular, on the use of the MYZONE technology, which is rapidly expanding around the world (i.e., more members are using WFT every day). Furthermore, this study extends the current literature about WFT and provides insights for suppliers, managers, and members of fitness centres. More specifically, the importance of the WFT goes beyond its physical aspects, as it also helps to motivate its members to perform physical activity; encourages interaction between the members; fosters a sense of community; encourages members’ to stay enrolled in their club; and, consequently, increases the profitability of fitness centres. 

## Figures and Tables

**Table 1:** Mean, standard deviation, skewness, and kurtosis of the indicators of the WFTS.

Items	M (SD)	Skewness	Kurtosis
1. Change in social interaction among members of MYZONE.	5.24 (1.8)	−0.77	−0.52
2. Change in social interaction among MYZONE members and fitness facility staff.	5.69 (1.5)	−1.13	0.78
3. Increase in sense of community among MYZONE members and fitness facility staff.	5.27 (1.6)	−0.85	−0.04
4. Oversharing of personal WFT physical activity data.	2.62 (1.7)	1.04	0.26
5. Use of various incentives to drive MYZONE members’ physical activity.	5.54 (1.5)	−0.97	0.25
6. Use of color-coded displays based on MYZONE members’ heart rates.	5.90 (1.5)	−1.7	2.45
7. Development of physical challenges based on MYZONE member data.	5.19 (1.7)	−0.75	−0.19
8. Addition of competitive elements to physical activity based on MYZONE member data.	5.30 (1.6)	−0.80	−0.02
9. Changes to the physical design and layout of fitness facilities to support WFT use	4.26 (1.8)	−0.24	−0.88
10. Fitness facility’s overemphasis on MYZONE-driven competition.	5.08 (1.7)	−0.72	−0.29
11. Members’ overemphasis on MYZONE-driven competition.	5.33 (1.5)	−0.90	0.26
12. Change in perceptions of accountability between MYZONE users and fitness facility staff.	4.34 (1.8)	−0.27	−0.84
13. Quantified physical activity metrics of MYZONE members.	5.63 (1.4)	−1.21	0.92
14. Fitness facility’s use of MYZONE data to increase members’ physical activity.	5.17 (1.7)	−0.77	−0.35
15. Fitness facility staff’s use of MYZONE data to increase users’ physical activity.	5.47 (1.8)	−1.12	0.30
16. Fitness facility’s and staff’s monitoring of WFT users’ activity beyond physical facility.	4.32 (2.2)	−0.21	−1.38
17. Fitness facility’s and staff’s use of WFT data to reduce/limit users’ physical activity	3.10 (2.1)	0.57	−0.95
18. MYZONE members’ overreliance on objective output proven by MYZONE devices.	5.87 (1.2)	−1.34	1.96

**Table 2:** Correlation analysis.

	1	2	3	4	5	6	7	8	9	10	11	12	13	14
**Item 1**	1.000													
**Item 2**	0.663	1.000												
**Item 3**	0.784	0.592	1.000											
**Item 5**	0.560	0.533	0.552	1.000										
**Item 6**	0.448	0.462	0.515	0.557	1.000									
**Item 7**	0.581	0.459	0.593	0.696	0.577	1.000								
**Item 8**	0.647	0.519	0.693	0.607	0.604	0.710	1.000							
**Item 10**	0.541	0.413	0.647	0.488	0.525	0.524	0.579	1.000						
**Item 11**	0.564	0.540	0.555	0.535	0.508	0.527	0.553	0.617	1.000					
**Item 12**	0.408	0.386	0.499	0.382	0.429	0.423	0.479	0.513	0.411	1.000				
**Item 13**	0.485	0.544	0.486	0.564	0.712	0.597	0.628	0.541	0.611	0.462	1.000			
**Item 14**	0.463	0.493	0.519	0.412	0.545	0.491	0.609	0.591	0.529	0.453	0.649	1.000		
**Item 15**	0.467	0.503	0.537	0.430	0.489	0.448	0.540	0.648	0.498	0.578	0.548	0.638	1.000	
**Item 18**	0.476	0.598	0.504	0.499	0.533	0.452	0.533	0.422	0.617	0.340	0.607	0.550	0.472	1.000

**Table 3:** Exploratory Factor Analysis.

Items	F1	Communalities
1. Change in social interaction among members of MYZONE.	0.75	0.57
2. Change in social interaction among MYZONE members and fitness facility staff.	0.70	0.49
3. Increase in sense of community among MYZONE members and fitness facility staff.	0.79	0.63
5. Use of various incentives to drive MYZONE members’ physical activity.	0.72	0.52
6. Use of color-coded displays based on MYZONE members’ heart rates.	0.73	0.53
7. Development of physical challenges based on MYZONE member data.	0.76	0.57
8. Addition of competitive elements to physical activity based on MYZONE member data.	0.82	0.68
10. Fitness facility’s overemphasis on MYZONE-driven competition.	0.74	0.55
11. Members’ overemphasis on MYZONE-driven competition.	0.74	0.55
12. Change in perceptions of accountability between MYZONE users and fitness facility staff.	0.60	0.36
13. Quantified physical activity metrics of MYZONE members.	0.78	0.61
14. Fitness facility’s use of MYZONE data to increase members’ physical activity.	0.73	0.53
15. Fitness facility staff’s use of MYZONE data to increase users’ physical activity.	0.70	0.49
18. MYZONE members’ overreliance on objective output proven by MYZONE devices.	0.69	0.48

Note: F1: EFA factor loadings; λ: CFA factor loadings.

## Data Availability

The data presented in this study are available on request from the corresponding authors.

## Appendix A

**Table A1:** The translation process of WFT items.

Social Interaction (4)	Interação Social (4)
***Member–member interaction***	***Interações membro–membro***
Change in social interaction among users of WFT.	Promove a interação social entre os sócios-membros que utilizam o MYZONE.
***Member–staff interaction***	***Interações membro–staff***
Change in social interaction among WFT users and fitness facility staff.	Promove a interação social entre os sócios-membros da MYZONE e os instrutores do ginásio.
***Community building***	***Construção de uma comunidade***
Increase in sense of community among WFT users and fitness facility staff.	Desenvolve o sentido de comunidade entre os sócios-membros que utilizam o MYZONE e os instrutores do ginásio.
***Over socialisation***	***Para além da socialização***
Oversharing of personal WFT physical activity data.	Partilha exagerada dos seus dados de treino na MYZONE.
**Gamification (6)**	**Gamificação (6/7)**
***Rewards and prizes***	***Recompensas e prémios***
Use of various incentives to drive WFT users’ physical activity.	Utilização de vários incentivos para motivar a atividade física dos sócios-membros da MYZONE.
***Colors***	***Cores***
Use of color-coded displays based on WFT users’ heart rates.	Utilização de mosaicos com cores com base nas frequências cardíacas dos sócios-membros da MYZONE.
***Challenges***	***Desafios***
Development of physical challenges based on WFT user data.	Criação de desafios físicos com base nos dados dos sócios-membros da MYZONE.
***Competition***	***Competição***
Addition of competitive elements to physical activity based on WFT user data.	Incremento de elementos competitivos à atividade física com base nos dados dos sócios-membros da MYZONE.
***Physical changes and displays***	***Alterações físicas das instalações e display***
Changes to the physical design and layout of fitness facilities to support WFT use.	Mudanças no espaço físico e design do ginásio para auxiliar os sócios-membros da MYZONE.
***Hyper competition***	***Híper competição (2)***
Fitness facility and/or WFT users’ overemphasis on WFT-driven competition.	Ênfase elevado do ginásio (para superar os objetivos) da competição MYZONE. Ênfase elevado dos sócios membros (para superar os objetivos) da competição MYZONE.
**Accountability (6)**	**Prestação de contas—mensuração (6/7)**
***Member-to-club accountability***	***Mensuração do membro para o club***
Change in perceptions of accountability between WFT users and fitness facility staff.	Alteração da responsabilidade entre os sócios-membros da MYZONE e os instrutores do ginásio.
***Quantification of physical activity***	***Quantificação da atividade física***
Quantified physical activity metrics of WFT users.	Medição/quantificação da atividade física dos sócios- membros da MYZONE.
***Increase physical activity*** Fitness facility’s and staff’s use of WFT data to increase users’ physical activity.	***Aumento da atividade física (2)*** O ginásio utiliza os dados da MYZONE para aumentar a atividade física dos sócios-membros. Os instrutores utilizam os dados da MYZONE para aumentar a atividade física dos sócios-membros.
***Monitor physical activity***	***Monitorização da atividade física***
Fitness facility’s and staff’s monitoring of WFT users’ activity beyond the physical facility.	O ginásio monitoriza a atividade física dos sócios-membros da MYZONE fora das instalações do ginásio.
***Limit physical activity***	***Limitar a atividade física***
Fitness facility’s and staff’s use of WFT data to reduce/limit users’ physical activity.	Os instrutores utilizam os dados do MYZONE para limitar a atividade física dos sócios-membros.
***Technology reliance***	***Confiança na tecnologia***
WFT users’ overreliance on the objective output provided by the WFT device.	Confiança dos sócios-membros nos resultados apresentados pelo dispositivo da MYZONE.
